# 

**Published:** 1876-04

**Authors:** 


### Books and Pamphlets Received.

A Treatise on Diseases of the Nervous System. By William A. Hammond, M. D., etc. Sixth edition, enlarged and improved. New York: D. Appleton & Co. 1876. Buffalo: Herger & Ulbrich.

Cyclopædia of the Practice of Medicine. Edited by Dr. H. Von Ziemssen. Vol. IV. Diseases of the Respiratory Organs. Albert H. Buck, M. D., American Editor. New York: Wm. Wood & Co. 1876,

A Series of American Clinical Lectures. Edited by E. C. Seguin, M. D. Vol. II, No. 2. On Certain Forms of Morbid Nervous Sensibility. By J. S. Jewell, M. D. New York: G. P. Putman's Sons.

### Medical and Surgical Journal

PUBLISHED MONTHLY,

Containing Original Articles, Reports of Medical Societies and Hospitals, Editorial, Reviews, Correspondence, Army News, etc., etc; including the usual variety of Medical Periodical Publications. Specimen copies sent on application

Address Buffalo Medical & Surgical Journal, Buffalo, N. Y.

TERMS OF SUBSCRIPTION, $3.00 PER YEAR, IN ADVANCE.

